# STRYV365 *peak team* and *Brain agents*: teacher perspectives on school impact of a trauma-informed, social–emotional learning approach for students facing adverse childhood experiences

**DOI:** 10.3389/fpsyg.2024.1388499

**Published:** 2024-10-08

**Authors:** Abbey Stoltenburg, Madison McGuire, Elizabeth Liverman, Paula Lumelsky, Garrett Bates, Constance Gundacker, Brandon Currie, John R. Meurer

**Affiliations:** ^1^Institute for Health & Equity, School of Medicine, Medical College of Wisconsin, Milwaukee, WI, United States; ^2^STRYV365, Milwaukee, WI, United States

**Keywords:** social–emotional learning, trauma-informed, childhood experiences, physical activity, video games, teacher perspectives

## Abstract

**Introduction:**

Adverse childhood experiences (ACEs) have a profound impact on children’s and adolescents’ academic performance and overall wellbeing. In contrast, positive childhood experiences help mitigate the negative effects of ACEs on health and wellbeing. Trauma-informed and social–emotional learning (SEL) programs in schools may address these impacts, with school staff playing a pivotal role in ensuring their success and influencing school outcomes. This study aimed to assess the strengths, weaknesses, and areas for improvement in the STRYV365 *peak team* and *Brain Agents* programs. The aim was to refine program implementation and determine the preliminary effects on students, teachers, and the broader school environment.

**Methods:**

To achieve this goal, four focus groups were conducted after the program concluded, involving 17 school leaders, administrators, and teachers from four urban schools serving students in fifth through ninth grades. The audio transcripts were professionally transcribed and analyzed by four co-authors using Dedoose for inductive coding, leading to the identification of major themes and subthemes.

**Results:**

Three key themes were identified from transcripts: school-wide perspectives of STRYV365 programs, strengths and impacts of STRVY365 programs, and suggestions for program improvement. School staff noted that the *peak team* was particularly engaging for students, helping them develop SEL skills and foster both peer-to-coach and peer-to-peer relationships. Additionally, the STRYV365 programs were found to enhance student–teacher relationships and staff relationships. Teachers expressed strong interest in taking a more active role in future programming.

**Discussion:**

Overall, this study highlights the importance of teacher and administrator involvement in maintaining consistent SEL activities for urban youth. The findings also suggest that short-term, 4-week SEL programs can have a positive effect on school culture, as well as on student–teacher relationships and teacher–teacher relationships.

## 1 Introduction

School environment and adverse childhood experiences (ACEs) significantly affect youth health and wellbeing, with childhood trauma linked to poor health outcomes, reduced school attendance, and behavioral issues (Christian-Brandt et al., 2020). ACEs include events such as having a family member incarcerated, witnessing or experiencing violence and abuse, or living with someone who has a substance use disorder, all of which can profoundly affect children’s development (Centers for Disease Control and Prevention, 2023).

Students who experience ACEs are more likely to face suspension, grade retention, chronic absenteeism, and lower academic performance compared to their peers without ACEs (Stewart-Tufescu et al., 2022; Webb et al., 2022). Black and Latino individuals are disproportionately affected by ACEs (Giordano et al., 2021), often living in stressful environments that shape their coping mechanisms and resilience (Sheffler et al., 2019).

Enhancing protective factors through positive childhood experiences (PCEs) can help mitigate the impact of ACEs, leading to improved health, wellbeing, and interpersonal relationships in the future (Frankowski, 2023; Huang et al., 2023). PCEs include fostering stronger neighborhood social interactions, increasing social engagement in schools, and promoting positive relationships between parents and children (Huang et al., 2023). Research shows that children with multiple positive experiences are more likely to experience better mental and physical health outcomes in adulthood compared to those with fewer positive experiences (Frankowski, 2023; Huang et al., 2023).

To address ACEs in students, programs have been developed to improve emotional regulation, develop coping strategies, and reduce stress to lower criminal the risk of behavior and address the deficits caused by ACEs (Giordano et al., 2021). By improving skills such as impulse control, optimism, goal-setting skills, and self-efficacy, these programs encourage better decision-making, leading to improved long-term health for youth (Logan-Greene et al., 2017). For African American youth dealing with parental dysfunction and poverty, participation in pro-social activities has been shown to strengthen coping and social skills, making them less reactive and hostile during conflicts (Logan-Greene et al., 2016).

Another method of mitigating the impact of ACEs involves trauma-informed programming, which trains staff to be sensitive to students’ trauma and to avoid re-traumatizing them. This type of programming has been shown to improve school engagement, reduce behavioral problems, and enhance social and emotional skills (Institute of Education Sciences, 2021; McInerney and McKlindon, 2014).

Additionally, social–emotional learning (SEL) addresses trauma and helps students better understand and manage their emotions (Hoffman, 2009). SEL focuses on self-awareness, self-management, and social mindfulness to help children make responsible decisions, achieve their goals, and build relationships (Hoffman, 2009). School-based SEL programs lead to positive attitudes toward self and others, positive behavior, academic success, and mental wellness (LaBelle, 2023; Li et al., 2011; Mahoney et al., 2018). SEL programs presented by teachers and school staff can be as effective as outside programming (Mahoney et al., 2018; Payton et al., 2008).

Interventions to improve students’ conflict-resolution skills, which can be underdeveloped in some adolescents, may help prevent future violence and enhance safety within schools.

Unfortunately, the COVID-19 pandemic saw a surge in violence within schools, affecting teachers, students, and other staff members. Violence in schools also affected teacher retention, with nearly half of teachers reporting they would consider leaving their jobs due to concerns about safety or school climate (McMahon et al., 2022). Improving school climate and fostering stronger school connectedness may enhance academic performance and student behaviors by strengthening interpersonal relationships between staff and students (Konold et al., 2018; Maxwell et al., 2017).

Teachers and school administrators play a crucial role in shaping the school environment, culture, and student outcomes.

Teachers view SEL as fundamental to student education (Dyson et al., 2019) and believe that developing social–emotional competence should take precedence over academic skills (Humphries et al., 2018). Their participation in SEL training is crucial, as it positively impacts their roles, sense of ownership, classroom dynamics, and student–teacher relationships (Lozano, 2021).

Greater support for a SEL culture in schools is a strong predictor of teacher commitment, with teachers being more willing to stay in schools that value the wellbeing of both students and teachers. Teachers’ comfort with SEL programming can also influence their level of stress, teaching efficacy, and job satisfaction (Collie et al., 2011; Collie et al., 2012). Given the critical role teachers play in student and school success, understanding their perspectives on SEL and trauma-informed programming is essential. This study aims to explore the views of teachers and school administrators regarding the STRYV365 *peak team* and *Brain Agents* programs, which incorporate SEL and trauma-informed programming to improve youth experiences and outcomes.

STRYV365 is a Milwaukee-based non-profit organization dedicated to building resilience and promoting positive life experiences for youth. It works toward this mission with its *peak team* and *Brain Agents* programs. The *peak team* (performance, empowerment, adaptation, and knowledge) program is a trauma-informed, coach-mentor-led initiative delivered during physical education classes, lunch, and/or classroom time. Its aim is to help students build resilience and recover from ACEs through mentoring, social–emotional support, and the creation of positive and fun activities to mitigate the negative effects of ACEs.

STRYV365 provides trauma-informed educational psychology training to its coach mentors, who have themselves overcome adversity, developed resilience, and are equipped to lead the program. The name of the program, *peak team*, is lowercase, which symbolizes the equal power dynamic between coach mentors and youth participants, emphasizing a TEAM approach that focuses on trauma, engagement, accessibility, and mentoring (Substance Abuse and Mental Health Services Administration, 2014). The *peak team* program attempts to create a safe environment, build a supportive community, promote trauma recovery, and employ empathy and child-centered methods to help youth build resilience.

Coaches are trained in SAMHSA’s six principles of a trauma-informed approach: safety, trustworthiness, and transparency, peer support, collaboration and mutuality, empowerment voice and choice, and cultural, historical and gender issues (Substance Abuse and Mental Health Services Administration, 2014).

Instead of following a rigid set of procedures, coach mentors apply these principles to guide their interactions with students. The program also uses the CRAFFT (Care, Relax, Alone, Forget, Family/Friends, and Trouble) framework (The Center for Adolescent Substance Use Research, 2018) and the COPE (Creativity, Optimism, Planning, Expert Information) change process to motivate students for behavioral change (American Psychological Association, 2011).

Coaches use CRAFFT-based mentoring strategies, including reviewing, recommending, risk advising, and responding to encourage youth to make self-motivational statements and reinforce their sense of self-efficacy (The Center for Adolescent Substance Use Research, 2018). The COPE framework helps STRYV365 structure educational materials and training manuals, with a focus on enhancing youth problem-solving skills through a systematic approach.

STRYV365’s *Brain Agents* is an interactive video game launched in the spring of 2020 by Night City, based on the frameworks of SEL and trauma-informed content. *Brain Agents* was created to expand the reach of STRYV365’s curriculum on mindfulness, problem-solving, and critical thinking to a broader audience, beyond those involved in *peak team* programming. This role-playing and action adventure video game is designed to empower youth by helping them navigate self-awareness, decision-making, and emotional self-regulation, as indicated by its capitalization.

The game aims to improve players’ openness, self-reflection, empathy, and tolerance of diverse perspectives—skills that have been proven effective in other SEL video game programs (Whitby and Kowert, 2022).

**Figure 1:**
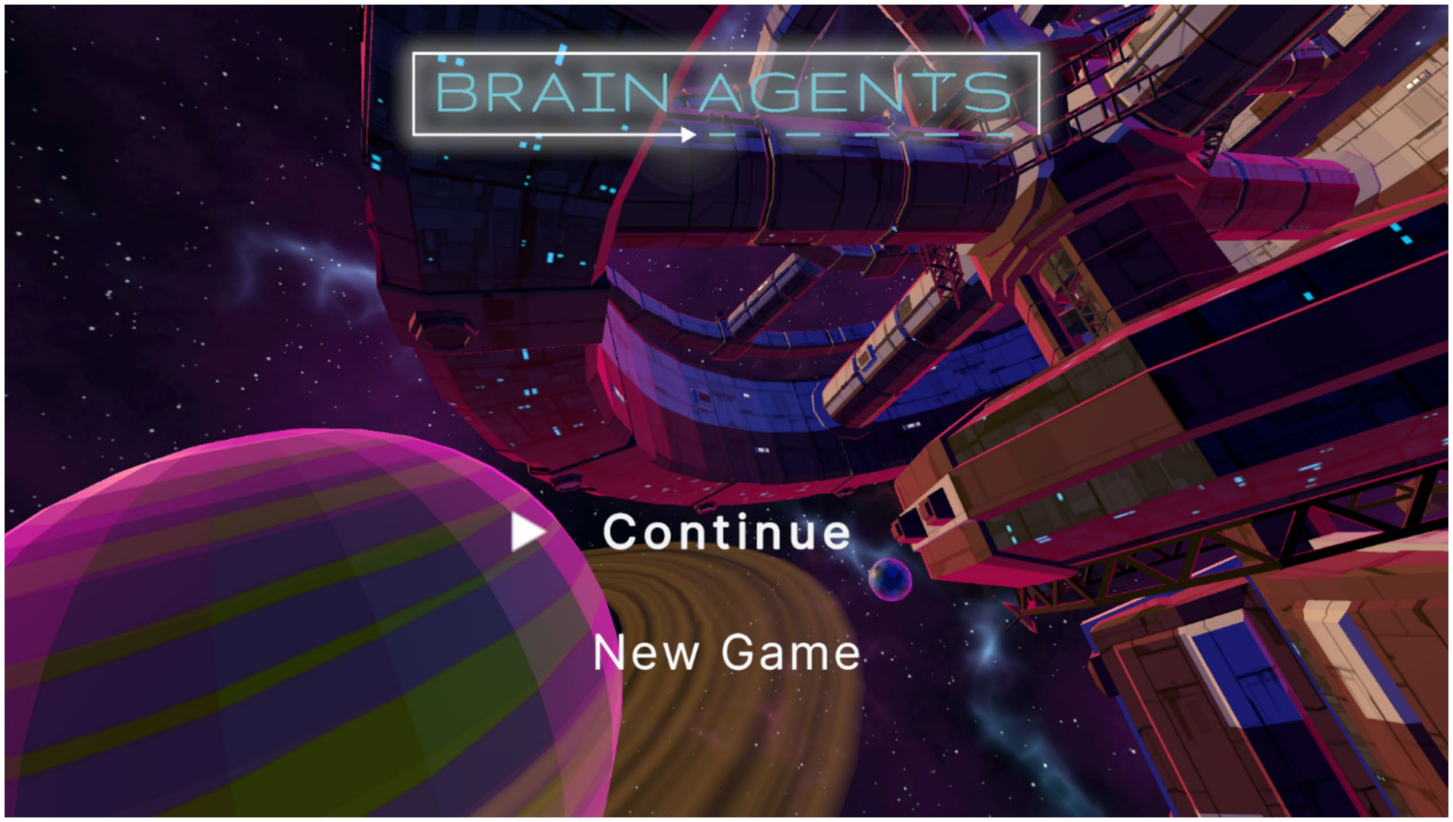
*Brain Agents* main game screen. A view of the players’ screen during gameplay.

**Figure 2:**
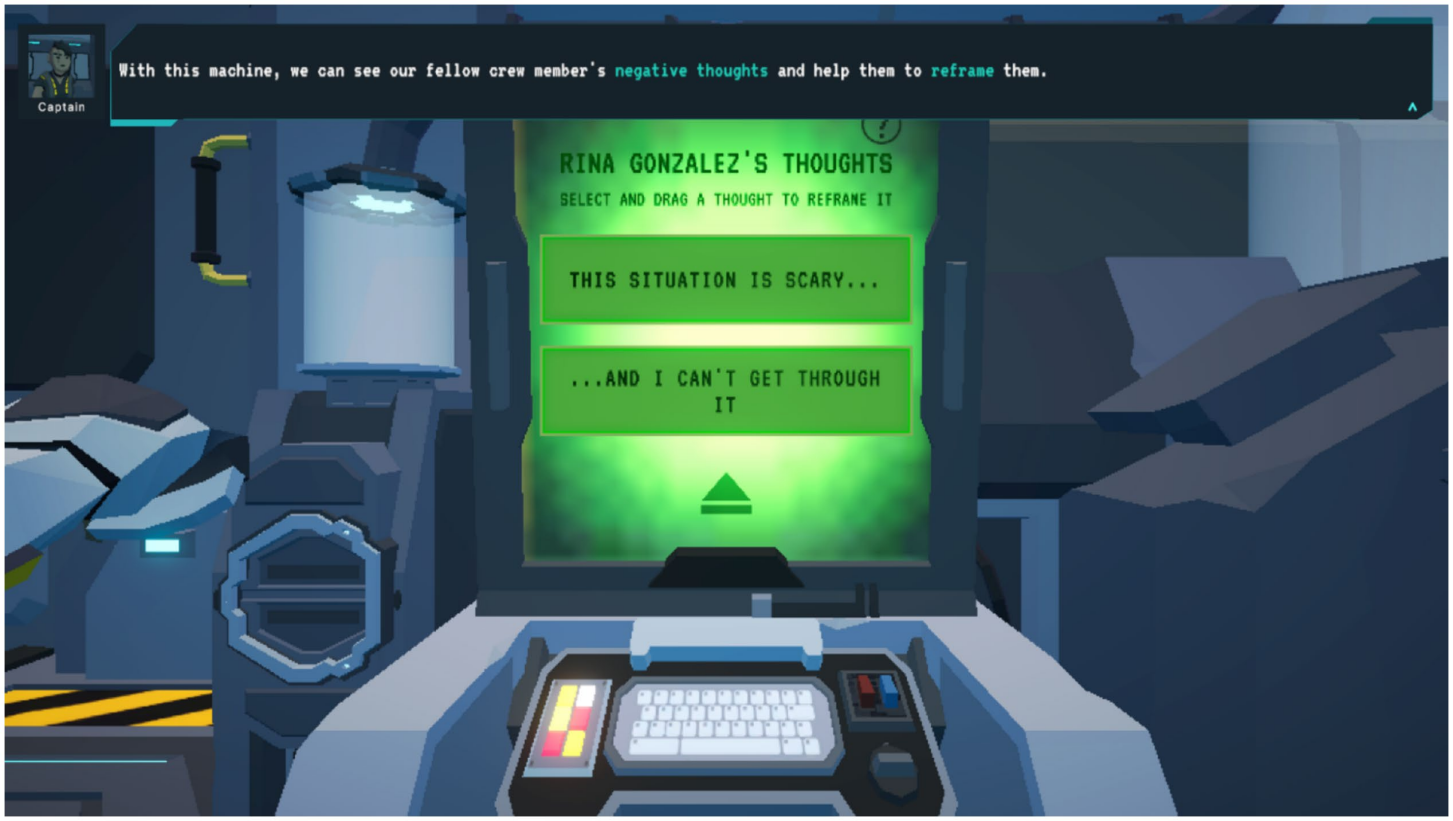
*Brain Agents* gameplay. Resilience-promoting messaging was displayed to participants during *Brain Agents* gameplay in the reframing cognitive distortions activity.

*Brain Agents* engages players in challenges across multi-level, scenario-based settings to foster resilient mindsets and support SEL among fifth to ninth-grade students in urban schools, through engaging and accessible digital game formats (Figure 1). Educational experiences are produced through narrative contexts, emphasizing the value of resilience, recognizing emotions and wellbeing, and establishing positive social relationships through the game’s storyline (Figure 2).

Minigames in *Brain Agents* help players learn by allowing them to practice coping techniques and problem-solving skills, understand the importance of work-life balance, identify and challenge cognitive distortions, recognize that current conditions can be improved through action, and connect with their emotions by linking physical sensations to feelings.

Additionally, these minigames foster social connections with in-game characters to reinforce teamwork concepts. *Brain Agents* delivers the STRYV365 learning curriculum to participants via Chromebooks or mobile devices, following an “early access” model of game development. As of this writing, *Brain Agents* is in active development and is not yet available to the public. Study participants can access the game through a designated website using a special codename assigned to them.

The purpose of this study is to present qualitative results from teacher and school administrator perspectives following the implementation of *peak team* and *Brain Agents* programs in four urban schools serving fifth- to ninth-grade students. The study aims to identify the strengths and weaknesses of the STRYV365 programs and assess their impacts on students, teachers, and the school environment. Additionally, opportunities for program improvement and potential expansion were evaluated.

## 2 Materials and methods

### 2.1 School participants and context

STRYV365 partnered with four urban schools, comprising two public and two private choice institutions, beginning in the 2022–2023 school year to offer programming. All schools had previously received some training in SEL and trauma-informed care, although individual teacher experience in these areas varied. The STRYV365 *peak team* program was piloted at one school during the 2021–22 school year, with both the *peak team* and *Brain Agents* programs expanding to all four schools in 2022–23.

A total of 1,626 fifth- to ninth-grade students attended the four schools. The demographic breakdown was as follows: 61% were Black/African American, 19% were White, 11% were Hispanic/Latinx, 6% were Asian/Hmong, and 3% were of mixed racial/ethnic background. Moreover, 69% of students were economically disadvantaged. Regarding performance in state standardized exams, 58% of students scored below basic, 27% were at basic, and 15% were proficient in English and language arts and mathematics. Furthermore, 28% of students were chronically absent, while 88% of students obtained high school diplomas.

### 2.2 Researcher qualifications and relationships (reflexivity)

The study was conducted from a positivist paradigm, using sociocultural theory to inform its conceptual framework for the study. This approach allowed the exploration of narrative and sense-making process through the participants’ and researchers’ voices, enabling the co-construction of data and its analysis. Reflexivity in the methodological approach allowed the co-authors to critically reflect on the choices made during data generation and analysis, ensuring that the participants’ subjectivity was valued. While the co-authors were older and had higher levels of education than the student participants, some shared similar racial/ethnic backgrounds and traumatic life experiences. All co-authors were involved in health and education career paths.

### 2.3 Conceptual framework and study design

The overall project conceptual framework hypothesizes that the *peak team* coaching and *Brain Agents* game interventions will influence students’ feelings, attitudes, behaviors, and school performance. During the 2022–24 academic years, a cluster randomized, incomplete block factorial design of interventions was developed across four schools and five grade levels (Table 1). Over the course of four semesters spanning 2 years, students in different classes were exposed to either the *peak team* program, *Brain Agents*, both interventions or no intervention each semester.

**Table 1:** Cluster randomized, incomplete block factorial design of interventions for 9th graders (excluding 5th through 8th graders and 2023–24 periods).

Data collection periods	School A	School B	School C	School D
Early fall 2022	None	None	None	None
Late fall 2022	None	*Peak team* and *Brain Agents*	None	*Brain Agents*
Late spring 2023	*Peak team*	None	*Brain Agents*	None

None means no prior program intervention, serving as a control group.

The *peak team* program lasted 4–10 weeks per semester, involving 9–12 sessions and 4–11 h of interaction. Students played *Brain Agents* at school two to three times per week for four to five weeks, completing an average of 10 sessions per semester, with each session lasting approximately 23 min—approximately 3.8 h of playtime. However, most students did not finish the entire game.

A 41-item survey assessing students’ feelings, attitudes, behavior, and perceptions of the interventions at six different time points will serve as the primary outcome measure (results to be reported in 2024). At the end of each programming semester, a random sample of consented students participates in focus groups or interviews to share their experiences.

### 2.4 Sampling, recruitment, consent, and assent

The Medical College of Wisconsin Human Research Review Board approved the research protocol. STRYV365 and school staff completed CITI training in human research protection. Parents of all 1,626 students were recruited via school events and emails to provide informed consent for their child’s participation in the study.

Students whose parents consented to their participation provided informed assent before completing the baseline REDCap survey (Vanderbilt, 2019) in October and November 2022. The full survey results are not included in this manuscript due to the ongoing nature of the study. The findings will be reported upon the completion of the 2-year evaluation. In May and June 2023, four in-person teacher focus groups were conducted with 17 participants, including teachers, school leaders, and administrators, with audio recordings obtained via Zoom.

### 2.5 Data processing

Professional transcription services were used to transcribe audio and media files from the focus group sessions. All data were securely stored on a Box drive. Deidentified data files were then exported into cloud-based Dedoose software (Dedoose, 2021) with password-protected access.

### 2.6 Data coding and analyses

Transcripts from the teacher focus groups were analyzed by four co-authors to identify themes and subthemes. Themes were initially identified using an inductive (top-down) approach based on participants’ responses to specific questions. The use of cloud-based Box software allowed multiple researchers to code transcripts simultaneously, which helped enhance intercoder reliability.

## 3 Results

### 3.1 Baseline summary of student participant surveys

Of the 399 parents who consented to their children’s participation in the study, 244 students completed the baseline REDcap survey. The sample included 244 students with 52% boys, 42% girls, and 6% identifying as other gender. The students reported experiencing various recent ACEs: 74% of students had someone close to them pass away, 56% of students had a close contact who was in jail, and 29% of students had someone close to them struggling with alcohol or drug-related issues. Moreover, 14% of students felt nervous or anxious daily, and 12% of them experienced feelings of sadness or depression on a daily basis. A school psychologist or counselor followed up with any student who showed wellbeing concerns. In terms of strengths and positive attributes, 83% of the students reported being satisfied with life and being aware of their emotions, 66% could calm themselves when upset, 63% reported no physical altercations or fights in the past 3 months, and 72% talked with friends about their problems.

### 3.2 Synthesis of main themes

From the interview transcripts with teachers and administrators, we identified three overall themes and corresponding subthemes, which were coded into three primary categories, each containing 4–6 subcodes. The three overarching themes were perceptions of the STRYV365 programming, the strengths and impact of the STRYV365 programming, and areas for improvement. Tables 2–4 provide examples of teacher and school administrator quotes illustrating these three main themes, drawn from focus groups held at the four schools participating in STRYV365’s *peak team* and *Brain Agents* programs.

**Table 2:** Theme 1: school perceptions of STRYV365 programs.

Subtheme: video game
“*Brain Agents* is … the online game that we did with students, the *peak team* is the targeted … interactive coaching and mentoring … with students. At the high school we did it through their Phys Ed classes.” “As far as *Brain Agents*, I know that … was a program … designed specifically for STRYV and it’s supposed to help students learn how to make more positive choices. I do appreciate the fact that we are able to play around with it so we can give our feedback, but we do know that ultimately kids learn a little better from playing games. I think that’s neat if you guys have something specific to your organization.”
Subtheme: student improvement
“I know that it is trauma-based programming … so teaching kids how to manage some of their emotions in different ways through sports and the gym classes and … now through *Brain Agents*. I think it’s really great to see that you guys are attacking it from different angles. Obviously, not everybody’s super athletic, so they might not be able to draw those examples from the gym class. Then, maybe they are going to get more out of *Brain Agents*.” “My understanding is that they both focused on STRYV’s mission of resilience … whether it was through the video game and obstacles that they might have to overcome through the video game and the *Brain Agents* or in person in the group, in the small groups … also focusing on resilience.”
Subtheme: program logistics
“My understanding was in our nest classes, which are like a homeroom, were the *Brain Agents*. We had different grade levels, different semesters, so the STRYV team would go into those … homeroom classes for the *Brain Agents* … usually once a week, and then in our gym classes … which meet every other day; the *peak team* or the game component focusing on resiliency.”
Subtheme: connection and community with schools
“I can just say they appreciate when y’all are here when they are working with you, they enjoy it.” “I know the kids are always excited when they have got STRYV for a special … but … to see the consistency throughout the years, the kids are like, “Oh yeah, you are that coach, you are that tall one,” and then, the tall one, that’s always, I think good for them.”

**Table 3:** Theme 2: strengths and impacts of STRYV365 programs.

Subtheme: learning social emotional learning skills
“I believe that … because I get them as freshmen, is that the freshmen, their inner child, is activated but they act like, I’m alone. … I feel like it allows them to have that social–emotional learning concept and because some of them are dealing more with real life, now that they are no longer in … grade school, now they are walking into adulthood slowly in high school, that they realize how to deal with emotions and other people.” “I like how you [STRYV365] tie the physical to the mental and the emotional. I think just based on our student body, we struggle with a lot of how can I cope, what can I do that will help me with the strong emotion that I’m feeling or process this mental, whatever, thinking.”
Subtheme: engaging physical activity and video game
“They do have Chromebooks all the time and we are always kicking them off of games, they found it interesting that … you guys are letting us play a game, and although it’s not the game we want to play, it is some kind of game. So, the gamification of the program was a highlight.” “They would come into my class once a week during the first semester and basically, as soon as they came in, the students were engaged with them. They did not have to do a call to attention. I did not have to get them to stop doing what they were doing. They wanted very quickly to be part of what was going on.” “I like how you all [STRV365] bring different activities besides basketball. No, let us open up their minds. It’s more than basketball and football, so I really enjoy that. … They like options.” “The idea of getting people moving, I feel like a lot of our kids are stuck in the house on the phone, on social media. I just like to see them move around and just go play, do not stand by me, go play, go play.” “I also think so much social emotional learning that people are putting into classrooms now is so … online based, so be able to have you guys here on site and not just the classroom stuff, not just the gym sets, changing it up often. It keeps the kids’ attention a lot more too.”
Subtheme: connection and forming relationships
“I think this year too, STRYV specials came in at a perfect time because I had been out for so long for a period and so for me to come back in and when I did not have that bond with my students anymore, the STRYV, especially in the classroom, helped rebuild that. It really forces them to work as a team. … What a perfect example of why we should have a year-round for everybody, right?” “I like those community building things. I feel like that always just helps the culture. … because during the day there’s a lot of stressful things going on. Sometimes, things get communicated poorly and people get frustrated with other people, but then it’s at the end of the day, yeah, we are just all human beings. Playing a goofy game, acting out pictures of someone bowling is really funny, and I think helps, has helped me build better relationships with teachers.” “I think … the staff really engaged well with the students. Students were excited, um, to see staff. They, when they saw them even outside of the classroom, they were given high fives and greeting them. … They really appreciated the energy that, um, all of the staff from STRYV365 brought into our building.”
Subtheme: STRYV365 coaches in *peak team*
“From my experience, the strengths have been engaging the students in learning they would not otherwise have because it’s not already embedded in our curriculum. Another benefit is getting them time away from the teacher, away from my voice all the time. It provides a variety and keeps things interesting.” “I think the strengths were competency of the staff. All the staff that I worked with were pretty skilled with, I do not want to call it intuition, but quickly picking up things with kids that we also know or also see, and it can be worrisome when you are bringing an outside group in to work with students and just that fear that they might not be good at connecting with kids or understanding the complexities of academics, home life, all the different things.” “The consistency, them being able to have STRYV from year to year I think is really important … we have lots of great partnerships and programs, but many of them are one or two grade levels, but for kids to be able to see the same faces and have the same mentors and facilitators from year to year and help build relationships …. That relationship building, I think, is foundational, especially for the kids in our building and a lot of the trauma that they are dealing with outside of our program.”
Subtheme: impact on school functioning
“The strength is just, I mean, being here since the beginning of the partnership and now, because at first we are like, “What is this,” and teachers and then getting it incorporated, getting us teachers involved and doing all the activities. It’s more buy-in for the kids because the teachers are seeing this is what they are talking about and we are able to bring it back in either during our SEL lessons or during our actual lessons. You’re going to get frustrated but think about all those strategies and things.” “I feel like it makes us work a little bit better when we were in meetings. One thing … and I, we always josh with the kids and we are like, “Well, when we are in our training, we are always on the winning team.”’
Subtheme: unsure of the impact
“Honestly, in terms of the *peak team* or the *Brain Agents*, for me what I saw that was logistics setting it up, going in there, seeing the kids interact, I did not sense that the *Brain Agents* was a big hit in the middle school. It was something the kids did. The nest [homeroom] teachers were more concerned about knowing if that person was coming or not and the timing of that, and if they were five minutes late, then they would be like are they coming ‘cause it’s only a 30-min window. So, the *Brain Agents* wasn’t a big hit I guess I would say.” “I do not know that I would have anything to add, um, to that. It was a rough start to the year … second semester was significantly better than first semester. So STRYV may have played some, some part of that, but I do not, I do not know that I would have data to defend that one way or the other.”

**Table 4:** Theme 3: opportunities for improvement.

Subtheme: teacher involvement
“For my own curiosity’s sake as a teacher, I would’ve liked to have had an in-class demo of some sort so I know what the students were doing so I could also engage and ask them questions about how, if it had any effect on their social emotional learning, what they got from it, what they liked about it. I would’ve liked to have been involved at least a little bit more on the onset.” “We have a specific group of teachers that are really knowledgeable and have had a lot of face time with STRYV, so we have got those folks that are buying in. As a school, I think we can all be better … I want you guys in front of more people so we can get more of the buy-in … The things you are teaching are so important. The more people we can get to buy in, the more successful our kids and the program will be too.” “I think too with the strength is just, I mean, being here since the beginning of the partnership and now, because at first we are like, ‘What is this?’ and teachers and then getting it incorporated, getting us teachers involved and doing all the activities. It’s more buy-in for the kids because the teachers are seeing this is what they are talking about and we are able to bring it back in either during our SEL lessons or during our actual lessons. You’re going to get frustrated but think about all those strategies and things.”
Subtheme: logistics
“So maybe an email package with a video saying, hey, this is what this is, this is what they’ll see, the student facing visuals for the *Brain Agent*s game. Maybe a timeline, some kind of project management-ish schedule … Also, just an intro of the person who would be coming into our class. Yes, we met briefly, but being able to sit down with them for about 10 or 15 min to discuss basically anything that I had on my mind that day about the program and a wrap up with the teachers as well at the end.” “I was also thinking too it should be whatever we do, it should be grade, grade-based level. I feel like sixth, seventh and eighth grade, they all need something different.” “I think sometimes, they learn it in the specials or whatever they do in the gym or something and then two or three months later, they take a survey on it and they are like, ‘What was that again?’ … if it was something like that where it’s weekly, a reminder of what STRYV does and who they are, I think that’s cool.”
Subtheme: communication about the research evaluation
“I was just going to with the video game, it would be interesting to see if you could track any changes in how they engage with it as time goes on … I’d be curious what metrics you are looking at maybe in how they answer questions, and those kind of things, as well. “Possibly getting a report of some sort at the end about student feedback, what they say they got out of it, and because I’m not seeing these kids on a daily basis, I cannot speak to differences in behavior, differences in handling of social emotional situation.”
Subtheme: expanding STRYV365 programming
“I mean, I think I wish we could have more time than just one special cycle or just one little bit of it. I wish it could be consistent like a regular class. I get it, but that would, I think, really be powerful too.” “I think a couple of you guys talked about de-escalation and physical and for me. My vision will be to have them in SEL and then you are getting those multiple intelligences, the physical, just looking at the screen or just talking because some kids just need to talk to somebody in the morning … I think giving them the choices too on how they start their day … Some kids, they come right in and doing some of those activities with STRYV would give them something positive as well as, like you said, do some de-escalation so they do not have as much built up by the time they do get to some of those situations.”
Subtheme: further applications of STRYV365 programs
“Having more exposure with for them as well would allow them to be able to deescalate. There are peak times of the year where we notice, especially in high school and probably across the board, you get these more infractions of these spikes. People start getting messy, the holidays, breaks, and even during the early parts of the year, even probably being during some summer PD [professional development], I do not know what y’all would go about that, but having you all be a little bit more present when those peak seasons come up because we have had a brawl at high school.” “Wonder with the rise of social media and it becoming such an issue, I think there’s a whole new level of trauma that is coming and has come from social media, whether it’s confidence, whether it’s bullying. I think that’s just another thing to consider.” “I think one thing too … but we got to remember too, all of them are affected, but that group got affected a little bit differently because they were after everything that happened in elementary school and they were COVID for two years, so they had no middle school experience to help them get socially adapted.”

### 3.3 Theme 1: perceptions of STRYV365 programs

Teachers and school administrators observed that the STRYV365 programming had a game-like quality, which they felt effectively engaged students while fostering resilience and emotional self-management skills. They were aware of the school-specific logistics concerning student interactions with the STRYV365 coaches and noted the enthusiasm students showed for the STRYV365 programs (Table 2).

### 3.4 Theme 2: impact of STRYV365 programs

Teachers and school leaders shared that the STRYV365 *peak team* program was highly engaging for students, helping them develop SEL skills and build connections with their peers. The program also positively influenced student–teacher relationships, teacher collaborations, and the overall school culture. Teachers recognized the importance of engaging students in SEL, noting that it is “not typically embedded in [the] curriculum.”

They appreciated STRYV365 coaches’ consistency and their deep understanding of urban youth experiences. Some teachers even incorporated STRYV365 strategies into their class lessons and SEL instruction. However, some school staff members expressed uncertainty about the impact of the *Brain Agents* game, citing a lack of student engagement. Additionally, other administrators felt they could not assess the program’s behavioral impact without further research results (Table 3).

### 3.5 Theme 3: opportunities for program improvement

School staff members expressed a desire for greater involvement in future STRYV365 programs to enhance both “teacher and student buy-in” and to help schools better understand the impact of the services. They felt that more consistent and regular information sessions with staff would enhance program effectiveness. Schools overwhelmingly requested more STRYV365 programming focused on SEL skill-building and desired more time spent with students. Teachers also suggested incorporating intervention, particularly “de-escalation” strategies that could be used in schools to prevent or reduce fights.

**Figure 3:**
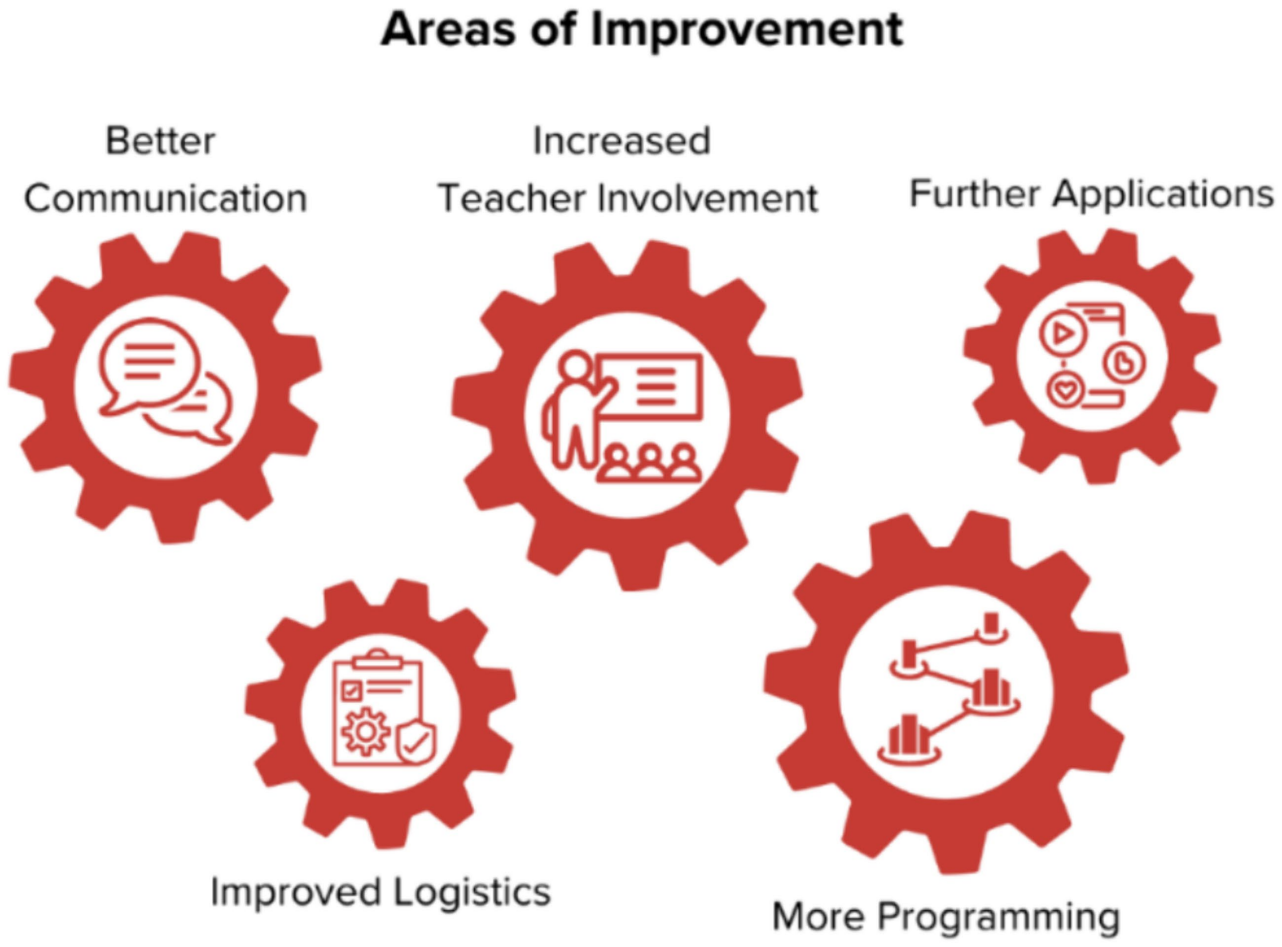
Opportunities for STRYV365 program improvement. Five areas of improvement were suggested: better communication, increased teacher involvement, expanded application of STRYV365 lessons within classrooms, improved logistics, and more programming offered by STRYV365.

The teachers noted that fights had become more common following the COVID-19 pandemic and expressed a need for more strategies to help students manage their emotions without resorting to violence. Additionally, they observed rising tensions between students on social media and recognized the need for more resources to address these online conflicts in school settings (Table 4). Based on the feedback from teacher and school administrator focus groups, Figure 3 depicts five areas for improving STRYV365 programming in schools: better communication, increased teacher involvement, expanded applications, improved program logistics, and more programs offered to schools.

## 4 Discussion

### 4.1 Summary of key findings

This qualitative study aimed to identify the strengths of the STRYV365 *peak team* and *Brain Agents* programs, as well as their impacts on students, from the perspectives of teachers and school administrators.

It also explored suggestions for improving STRYV365 programs. Based on focus groups with school staff, STRYV365 programs, especially the *peak team*, were found to be highly effective for students. However, some school administrators, whose involvement was limited to logistical scheduling, were uncertain about the program’s impact or felt that *Brain Agents* was less successful than the *peak team*.

Teachers who were more actively involved in student programming found the programs fun and engaging, allowing students to participate in healthy physical activities, and acknowledged the power of gamification in reinforcing lessons taught by STRVY365 coaches. Teachers appreciated the outside perspective provided by the coaches and valued the SEL skills being taught. They expressed interest in learning more about STRYV365 programs to better integrate the curriculum into their own lessons. Additionally, school staff felt the programs positively influenced their professional relationships and teamwork with colleagues.

Teachers expressed a desire for more involvement in the programs and suggested embedding STRYV365 activities into daily school routines. They also recommended expanding the programs to address social media conflicts and cyberbullying among students. Overall, both teachers and administrators valued the STRYV365 *peak team* and advocated for the expansion of services in their schools. These findings are preliminary, with the results of the 2-year study forthcoming.

### 4.2 Comparison with other similar interventions

Consistent with the findings of Giordano et al. (2021), which highlighted elevated ACE risk among African American and Hispanic youth, there was a high rate of ACEs among students in the four participating schools, which predominantly taught children of color.

Some students were already experiencing the impact of ACEs, as evidenced by 14% of students reporting daily feelings of nervousness or anxiety and 12% of them feeling down or depressed. Teachers described the STRYV365 programs as fun and engaging, aligning with the literature that highlights the benefits of gamification (Cejudo et al., 2020).

Similar SEL and trauma-informed programs have demonstrated positive outcomes, including increased student engagement, academic achievement, mental wellbeing, and reduced behavioral issues (Institute of Education Sciences, 2021; LaBelle, 2023; Li et al., 2011; Mahoney et al., 2018). However, in this study, discussions regarding specific SEL skills developed by students were limited.

While teachers expressed that they appreciated the SEL programming and believed students were learning to better manage their emotions, they did not provide concrete examples of when these behaviors were observed. It remains unclear whether this lack of specific examples is due to minimal behavioral changes in students or a lack of direct observation of the programs by the teachers.

Teachers expressed appreciation for the coaching and mentoring provided by the *peak team*, highlighting the respect students had for the coaches and the coaches’ competence in interacting with students. Previous studies have shown that school staff can effectively implement SEL programs and may have a greater impact than external personnel (Payton et al., 2008). However, this study does not evaluate whether using teachers instead of external coaches would enhance the program’s effectiveness. The *peak team* program is distinctive in its “coach” model, which focuses on building youth relationships with mentors in order to promote social–emotional development in children (Yeager, 2017).

Teachers expressed a desire for greater involvement in STRYV365 programs to better understand what students were learning and to incorporate program concepts into their curriculum. Previous studies have shown that teacher buy-in to programs enhances the effectiveness of trauma-informed services, and increased teacher education contributes to improved student wellbeing (Brunzell et al., 2019).

Enhancing teacher awareness or “buy-in” of STRYV365 programming could be a helpful strategy for amplifying the program’s impact on both participating students and schools. As teachers interact with trauma-informed programs, they shift their ideas and practices to better address classroom adversities in educational topics (Brunzell et al., 2019).

Teachers who were actively involved in STRYV365 programs noted significant benefits, such as incorporating SEL into daily classroom activities, fostering better student relationships, and improving overall school functioning. This engagement aligns with the SAMHSA principle of empowering teachers to meet students’ needs (Substance Abuse and Mental Health Services Administration, 2014) and research showing that teacher participation in SEL programs enhances classroom dynamics and strengthens student–teacher relationships (Lozano, 2021). While STRYV365 does not directly address cyberbullying, the focus on building self-management and relationship skills aligns with other SEL programs that have been shown to reduce bullying victimization (Yang et al., 2020).

### 4.3 Limitations of the approach and findings

The subjective nature of qualitative data analysis may have been influenced by the perspectives of coders and researchers, although the use of multiple coders helped mitigate potential biases. The presence of STRYV365 staff during the teacher and school administrator focus groups could have influenced responses. However, participants were candid in discussing both the strengths and weaknesses of the program.

The relatively small sample size of focus group participants limits generalizing the findings to the broad perspectives of all school personnel. Moreover, the level of participant engagement with STRYV365 programs varied, which may have affected the depth of their feedback. Variability in the success of coaches forming strong relationships with students may have also influenced the outcomes of the *peak team* program. The quantitative student survey results will be reported upon the completion of the 2-year project.

### 4.4 Implications for students, teachers, and schools

By observing and reflecting on the STRYV365 programs, teachers and school administrators appreciated the importance of student engagement, gamification, and teaching core concepts to foster learning. This study highlights the importance of teacher support in implementing SEL activities and the need for consistency, especially for urban youth. SEL programs such as STRYV365 have the potential to positively impact school culture, strengthen student–teacher relationships, and enhance collaboration among teachers.

In the wake of the COVID-19 pandemic, teachers expressed a strong need for ongoing support to develop SEL skills, which are essential for both students and educators. STRYV365 programs may encourage teachers to integrate more SEL-focused content into their lessons. By teaching coping strategies through programs like *peak team* and *Brain Agents*, schools can help prevent or resolve conflicts among students. Additionally, teachers can apply strategies learned from STRYV365 to cultivate stronger, more meaningful relationships with their students.

### 4.5 Conclusion

Feedback and suggestions from school personnel and students will guide future improvements in STRYV365 programs and refine research methods. STRYV365 also plans to expand its services through a “train the trainer” initiative, equipping teachers and school staff with trauma-informed and SEL principles to foster student resilience and promote positive life experiences. *Peak team* coaches will specifically address cyberbullying, and related questions will be incorporated into student surveys, focus groups, and interviews. Over the next year, student surveys assessing feelings, attitudes, and behavior, along with qualitative data, will be analyzed and reported as part of the 2-year evaluation.

In conclusion, this study suggests that even a brief, 4-week SEL program can positively impact student behaviors and school culture. Although not the primary objective, the intervention also led to improvements in teacher-to-teacher interactions, as noted by the participating teachers.

## Data Availability

The original contributions presented in the study are included in the article/supplementary material. Further inquiries can be directed to the corresponding author.
